# Supplementary material for: Small Heat Shock Proteins Collaborate with FAIM to Prevent Accumulation of Misfolded Protein Aggregates
Source: Int J Mol Sci. 2022 Oct 6;23(19):11841. doi: 10.3390/ijms231911841 (PMC9570119; doi:10.3390/ijms231911841)
Supplement: Supplementary file 1 [file ijms-23-11841-s001.zip › ijms-1836275-supplementary-final.pdf]

```
C. elegans Hsp-25   MPTYTRTYSYSKREMSERRIDVNRSNYSVIDN-EFGNMRDRFEQEMRRVEEEMKRLRSEF   59
H. sapiens HSP27    --------------MTERRVP-----FSLLRGPSWDPFR---------------------   20
                                  *:***:      :*:: . .:. :*

C. elegans Hsp-25   EGYRPNGGPPAAISNQPYNA--YSNTSSHHETSNRTGGFGSPLPPPSFHGPSDLMAHRPT   117
H. sapiens HSP27    -DWYPHS----RLFDQAFGLPRLPEEWSQWLGGSSWPGYVRPLPPAAIESPAVA---APA   72
                     .: *:.     : :* :.     :  *:   ..   *:  **** ::..*:      *:

C. elegans Hsp-25   YDPYLD-NLKSPLIKDESDGKTLRLRFDVANYKPEEVTVKTIDNRLLVHAKHEEKTPQR-   175
H. sapiens HSP27    YSRALSRQLSSGVSEIRHTADRWRVSLDVNHFAPDELTVKTKDGVVEITGKHEERQDEHG   132
                    *.  *. :*.* : : .  ..  *: :** :: *:*:**** *. : : .****:  ::

C. elegans Hsp-25   TVFREYNQEFLLPRGTNPEQISSTLSTDGVLTVEAPLPQLAIQQ----------------   219
H. sapiens HSP27    YISRCFTRKYTLPPGVDPTQVSSSLSPEGTLTVEAPMPKLATQSNEITIPVTFESRAQLG   192
                     : * :.::: ** *.:* *:**:** :*.******:*:** *.

C. elegans Hsp-25   -------------    219
H. sapiens HSP27    GPEAAKSDETAAK    205
```

Supplementary Figure S1. Alignment of *C. elegans* Hsp-25 and *H. sapiens* HSP27 sequences from publicly available databases. The protein sequences were aligned using the Clustal Omega program. Asterisks (*) denote single, fully conserved residues. Colons (:) denote conservation of strong groups, and periods (.) denote conservation of weak groups. No symbol indicates no consensus.

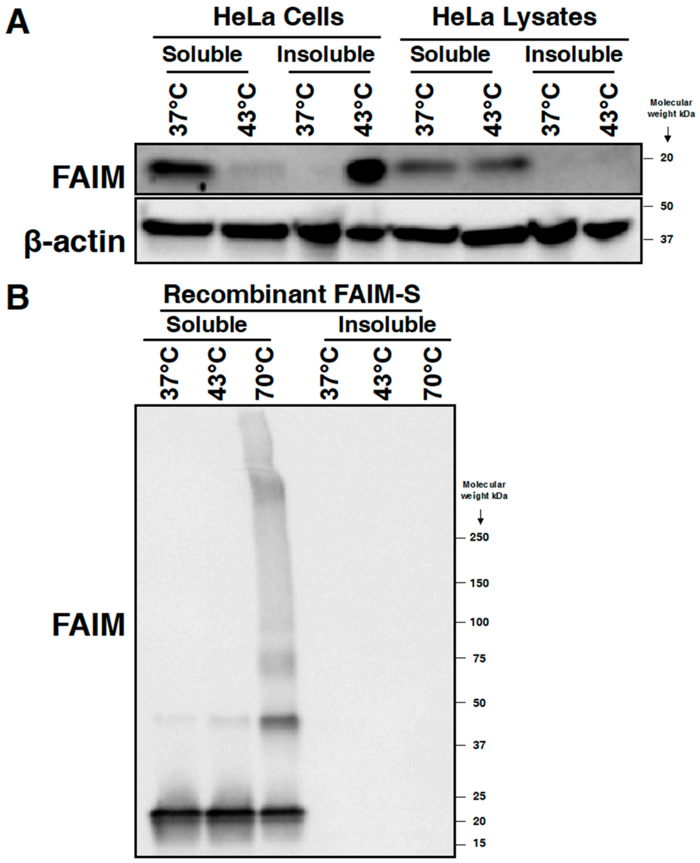

Supplementary Figure S2. FAIM protein shifts to insoluble in a cell-intrinsic manner. **A.** HeLa cells or HeLa cell RIPA lysates were incubated at 37°C, or were exposed to heat shock (43°C) for 2 hours, as indicated. Cells were then harvested and lysed in the RIPA buffer. **B.** 1uM of FAIM-S protein in RIPA buffer was incubated at 37°C, 43°C, or 70° C for 2 hours. Soluble proteins were isolated using RIPA buffer and RIPA buffer-insoluble proteins were extracted. Equal amounts of protein were analyzed by western blotting. Representative data are shown. Similar results were obtained from 3 independent experiments.

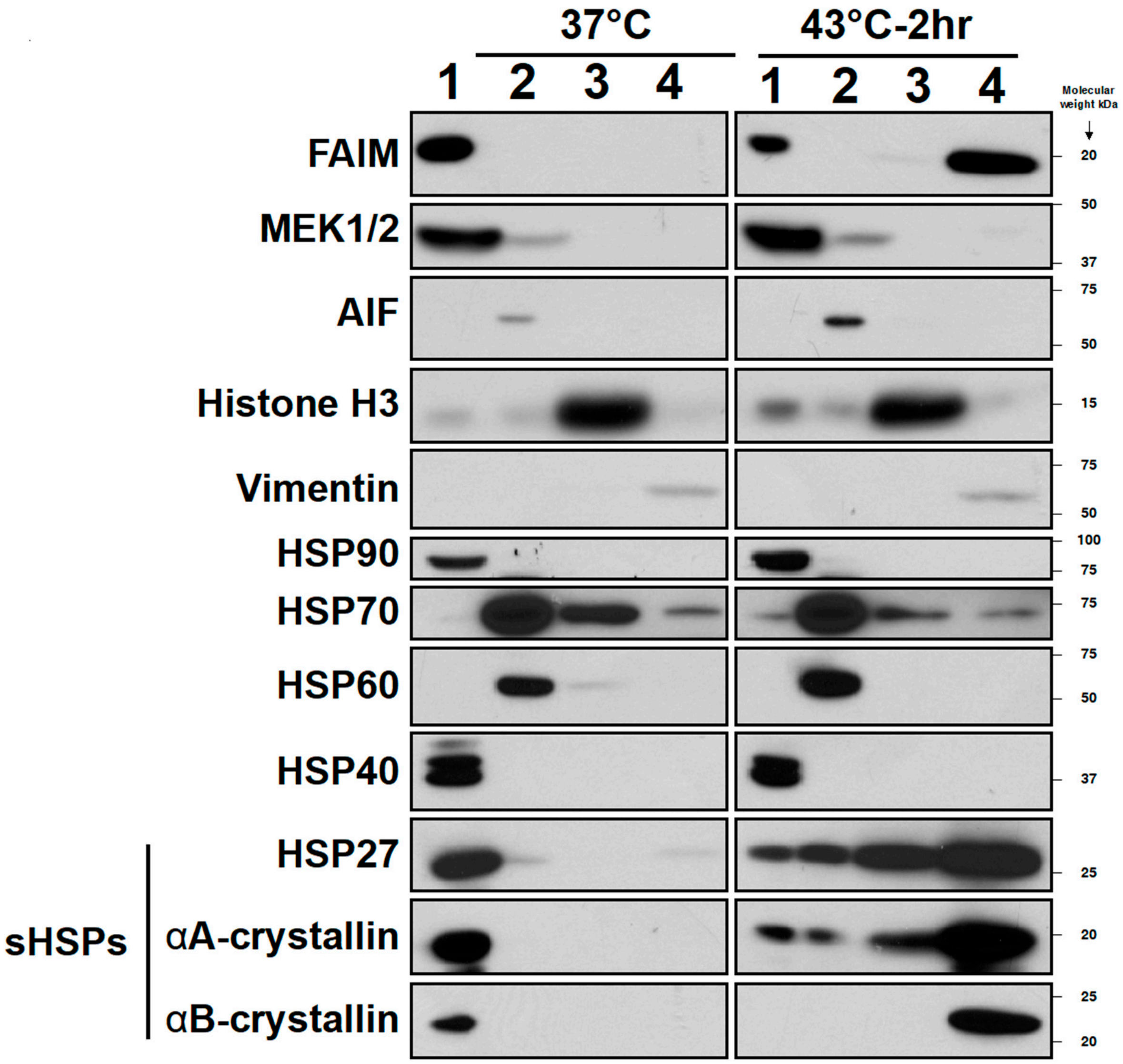

Supplementary Figure S3. FAIM protein shifts to the vimentin positive, cytoskeletal/detergent-insoluble fraction after heat shock in HEL B-3 cells. HLE B-3 cells were incubated at 37°C, or were exposed to heat shock (43°C) for 2 hours, as indicated. Cells were then harvested and proteins were divided into 4 fractions, 1; cytosol (MEK1/2-containing), 2; membrane/organella (AIF-containing), 3; nucleus (histone H3-containing) and 4; cytoskeleton/insoluble (vimentin-containing). Equal amounts of protein were analyzed by western blotting. Representative data are shown. Similar results were obtained from 3 independent experiments.

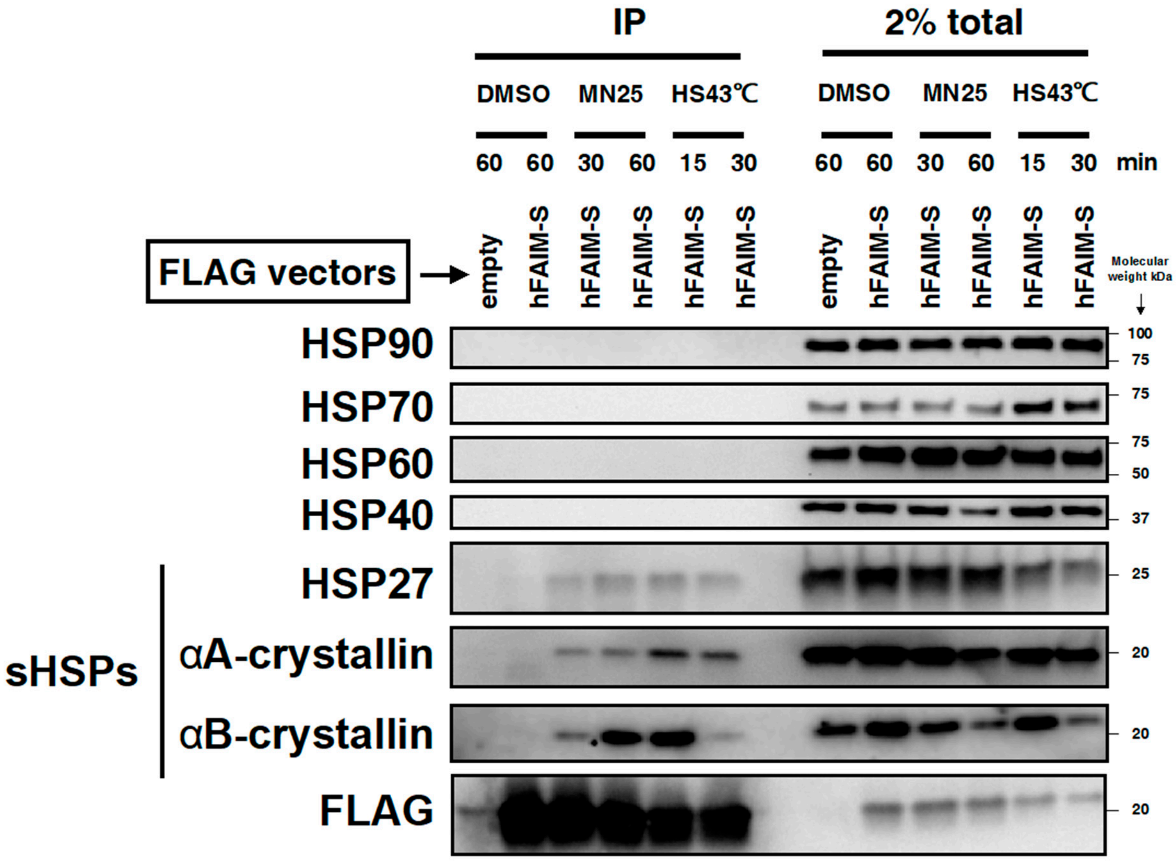

Supplementary Figure S4. FAIM associates with small HSPs upon Cellular Stress FLAG-tagged FAIM-S was transiently transfected into FAIM-KO HLE B-3 cells. Two days after the transfection, cells were harvested with or without stress induction provided by incubation with menadione (MN) at 25 mM or heat shock (HS) at 43°C for the indicated times, and lysed in RIPA buffer. Samples were immunoprecipitated with anti-FLAG and then western blotted for HSP proteins. Similar results were obtained from at least 3 independent experiments.
